# Strategies to improve healthcare team communication structure and quality in resource-variable childhood cancer hospitals (TeamTalk): a study protocol

**DOI:** 10.1186/s43058-025-00811-z

**Published:** 2025-11-17

**Authors:** Asya Agulnik, Dylan E. Graetz, Bobbi J. Carothers, Jocelyn Rivera, Erin Abu-Rish Blakeney, Samantha Hayes, Veronica L. Chaitan, Leopoldo Cabassa, Charles W. Goss, Douglas A. Luke, Sara Malone

**Affiliations:** 1 Department of Global Pediatric Medicine, St. Jude Children’s Research Hospital, Memphis, TN USA; 2 School of Public Health, Washington University in St. Louis, St. Louis, MO USA; 3 Department of Pediatric Emergencies, Hospital Infantil Teleton de Oncologia, Queretaro, Mexico; 4 Department of Biobehavioral Nursing and Health Informatics, School of Nursing, University of Washington, Seattle, WA USA; 5 Brown School, Washington University in St. Louis, St. Louis, MO USA; 6 School of Medicine, Washington University in St. Louis, St. Louis, MO USA

**Keywords:** Pediatric cancer, Implementation science, Low- and middle-income countries, Resource-poor settings, Social Network analysis, Interdisciplinary communication, Interprofessional communication, Teamwork

## Abstract

**Background:**

Healthcare team communication is essential to high-quality childhood cancer care, especially during high-acuity events such as clinical deterioration and in resource-variable settings, where supportive interventions to resolve deterioration are less available. Communication quality has traditionally been understudied in these settings, and there is a notable lack of communication interventions that are appropriate and feasible in settings across resource levels. We propose addressing this challenge in this study protocol, which will co-develop and pilot a multi-level intervention to improve communication and outcomes for children receiving cancer treatment.

**Methods/design:**

This study leverages systems and implementation science methodologies to evaluate and improve communication quality in the care of hospitalized children with cancer. We will use a newly developed reliable and multilingual measure of communication quality during clinical deterioration (CritCom). In this study, we will: 1) evaluate the relationship between healthcare team communication structures (using social network analysis) and quality (using CritCom) in the care of children with cancer, with a specific focus on the impact of hierarchy and modifiable communication determinants. We will then: 2) co-develop a multilevel intervention to address challenges in communication quality across variably resourced settings, using semi-structured interviews among clinicians working in these settings and intervention mapping with a global expert panel. Finally, we will 3) test the feasibility, acceptability, appropriateness, and preliminary efficacy of this novel intervention using a cluster-randomized wait list control pilot trial in eight resource-variable hospitals providing childhood cancer care with poor team communication quality.

**Discussion:**

This project identifies modifiable determinants of communication before co-developing and testing interventions with clinicians. When completed, this study will produce an evidence-informed, multilevel intervention to improve healthcare team communication during clinical deterioration, advancing the science of team communication during cancer care, and ultimately improving survival for children with cancer.

**Trial registration:**

ClinicalTrials.gov Record NCT07083674.

Contributions to the literature

- This work will provide a foundational understanding of modifiable determinants of communication that impact healthcare delivery in resource-variable settings.
- We will develop and test an intervention to improve healthcare team communication, a field that currently lacks effective interventions.
- We will use social network analysis, a systems science method, to describe structural variation in communication and compare it to organizational communication quality.
- This study will address two notable barriers to team communication during patient deterioration: (1) lack of understanding of specific modifiable determinants of communication structure and quality and (2) lack of appropriate, feasible, and measurable communication interventions.

## Background

To reduce global disparities in childhood cancer survival, the World Health Organization (WHO) Global Initiative for Childhood Cancer [1] and others [2] emphasize the need to improve childhood cancer treatment globally. Many hospitals providing childhood cancer care, however, face a range of resource limitations, including limited staff, supplies, and medications, challenging their ability to provide high-quality childhood cancer care and achieve this imperative [3]. A major barrier to childhood cancer survival, particularly in low-resource settings, is treatment-related mortality, or death during cancer treatment [4]. Clinical deterioration, defined as a decline in a patient’s clinical state that requires rapid identification and intervention, is a common cause of morbidity and mortality in children with cancer [5, 6]. Our prior work demonstrated a 30% mortality among pediatric oncology patients with deterioration in low-resource hospitals [7–13]. To date, however, there are limited effective interventions to improve outcomes for children with cancer who experience deterioration in resource-variable hospitals [14].

Optimal outcomes during clinical deterioration in children with cancer require excellent care coordination across multiple teams, necessitating high-quality team communication [15]. Over 70% of treatment delays, preventable harm, and death occur due to poor communication [16–23]. Prior studies have demonstrated that better communication leads to safer care, fewer medication errors [24], higher rates of rescue [25], and increased consistency in patient care across clinical teams [26]. High-quality communication between clinicians (e.g., physicians, nurses, pharmacists, respiratory therapy, etc.) directly impacts how teams utilize available resources and skills to identify and manage deterioration, thereby preventing mortality and improving health outcomes [27, 28]. Deterioration events, however, are highly stressful for clinical team members due to high patient acuity and time pressures, resulting in a risk of ineffective communication and lack of psychological safety, conceptualized as having a sense of safety in speaking up [16, 22]. Low-resource settings face additional communication challenges, including hierarchical systems, lower staff-to-patient ratios, and varying roles and responsibilities [29]. Addressing healthcare team communication, especially during acute events like clinical deterioration, offers a theoretically effective, low-cost intervention to improve childhood cancer outcomes across different settings. Previously studied interventions to improve pediatric oncology communication, however, focus on planned, controlled settings such as rounds or tumor boards and have been primarily developed and tested in high-resource settings [19, 30–33]. There is an urgent need for effective communication interventions to target complex systems and subsequently improve outcomes related to deterioration.

To improve communication in the care of children with cancer, we must identify modifiable factors that affect the quality of team communication [16, 21, 34, 35]. **“**Team communication” in this context must focus on the process of information exchange among the entire healthcare team, encompassing the quality of this exchange both between clinicians of different *professions* (physicians, nurses, respiratory therapist, social workers, etc.) and different *disciplines*(oncology, intensive care, surgery, etc.). However, there is a lack of a robust understanding of modifiable components of healthcare team communication structure and quality that can be targeted with interventions. Few effective interventions improve team communication and those that exist were primarily developed in high-resource settings [19, 30–33]. Low-resource settings are understudied [36], creating disparity between the high burden of childhood cancer mortality and poor understanding of communication determinants in these settings. This highlights a need for co-developed communication interventions appropriate for resource-diverse settings [37–39].

To address this knowledge gap, the TeamTalk study will develop and test a multilevel intervention that focuses on modifiable determinants of healthcare team communication structure and quality in global pediatric oncology hospitals, aiming to improve childhood cancer outcomes*.* To achieve this goal, we will 1) evaluate the relationship between team communication structure and quality in the care of children with cancer, 2) co-develop a multilevel intervention to improve communication quality in resource-variable hospitals, and 3) test the feasibility, acceptability, appropriateness, and preliminary efficacy of this intervention in resource-variable hospitals through a pilot cluster-randomized wait list control trial.

## Methods and design

TeamTalk is an observational, formative study of team communication in resource-variable pediatric oncology hospitals, followed by intervention co-development and pilot testing (Table 1). We will engage members of the global oncology community to assess the relationship between team communication structure and quality (Aim 1) and use a mixed-method approach to co-develop and test an intervention to address these challenges across settings (Aims 2/3).

**Table 1:** Overview of study aims

Overall Question: What are common modifiable determinants of healthcare team communication during deterioration and how might interventions target these determinants to improve outcomes for children with cancer in resource-variable settings?		
Aim 1: Communication structure and quality	Questions	What is the relationship between structure and quality of team communication around deterioration in children with cancer?
	Method	Cross sectional social network analysis from 10 high- and 10 low-quality communication hospitals selected from previous cross-sectional data (total *n* = 20)
	Outcome	Empirical relationship between communication quality, communication structures, and provider and hospital level determinants
Aim 2: Develop intervention to improve communication	Question	What are potential multi-level interventions to address common modifiable challenges to communication quality across both high- and low- resource hospitals?
	Method	(1) Sequential mixed methods study using quantitative data from Aim 1 supplemented by qualitative interviews with clinicians and (2) implementation mapping with global experts to co-develop an intervention to address common communication challenges
	Outcome	Multi-level intervention co-developed and selected for pilot testing
Aim 3: Pilot trial of communication intervention	Question	Is the identified intervention effective to improve communication quality and provider and patient outcomes while being perceived as feasible and acceptable by providers?
	Method	Pilot identified multilevel intervention at 8 resource-variable hospitals using a wait list controlled, cluster randomized trial
	Outcome	Identify feasibility, acceptability, appropriateness, and preliminary efficacy, of intervention to improve communication quality during deterioration

### Conceptual framework

**Fig. 1:**
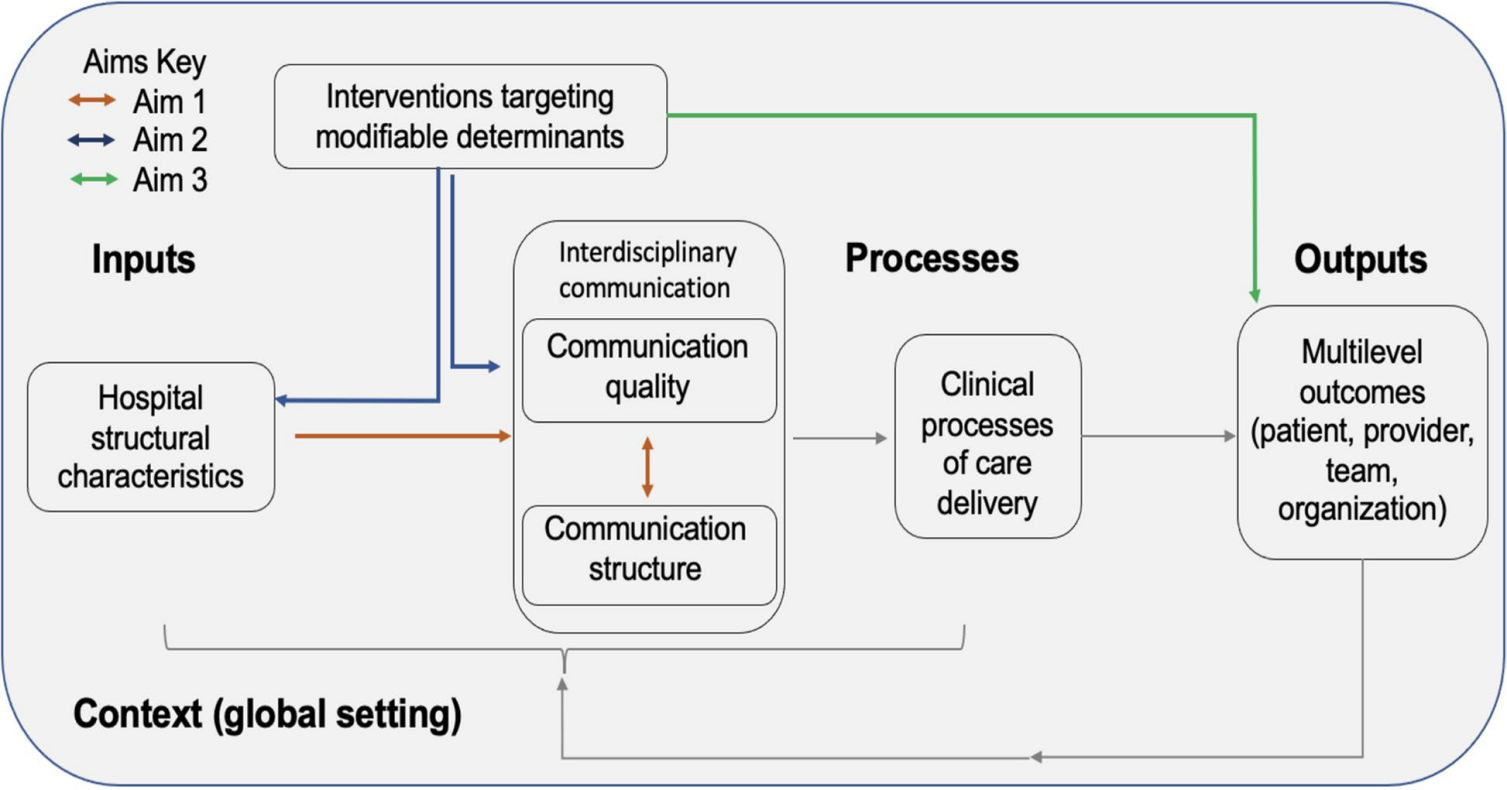
Overview of conceptual framework for this research, focusing on assessing communication structure and quality within resource-variable settings. This is adapted from the cancer Multiteam System (cMTS) framework

This study is informed by implementation science, health services research, and network communications theories, specifically the cancer Multiteam System (cMTS) framework (Fig. 1) [40, 41]. This conceptual framework illustrates how complex teams collaborate to deliver coordinated cancer care, highlighting the importance of effective communication structures and quality in care delivery. cMTS informs this study through its description of care processes and how teams are composed and organized, including team structure and hierarchy. Within the cMTS framework, communication is described as part of the processes of teamwork, while structure is an input. Care processes include both teamwork between and across teams as well as tasks that are accomplished through care delivery. These result in multilevel outcomes, including clinical patient outcomes, provider outcomes (burnout and job satisfaction), and unit/team outcomes (guideline adherence and efficiency of care) [40, 41].

**Fig. 2:**
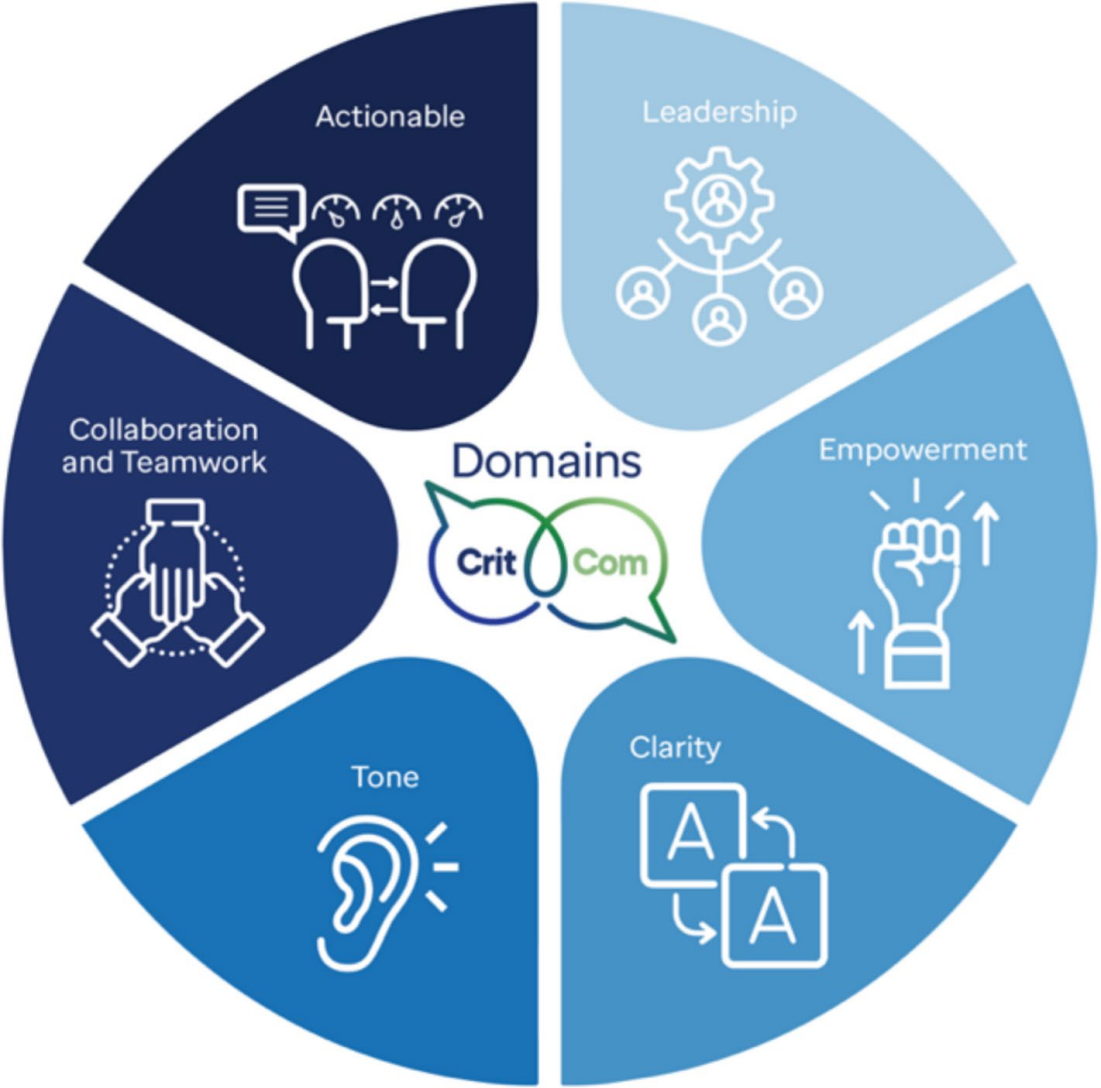
CritCom measure domains of quality team communication

Communication can be conceptually divided into two components: (1) quality of communication and (2) structure of communication [42, 43]. High-*quality* communication improves patient care, and we conceptualize this in alignment with the CritCom framework, a novel, reliable, multilingual measure of team communication quality comprised six domains (Fig. 2) [44, 45]. Less is known about how communication *structures*can promote enhanced care, although examples of this include hierarchies, standard communication norms, and escalation protocols. For this study, we conceptualize communication structure as informed by network theory. Network communication theory describes the structures of communication to understand individual interactions, feedback, and behavior constraints among those involved within complex systems [46–48]. While network theory views each individual actor as following fairly simple rules or behaviors, complexity occurs when these simple behaviors multiply into a system of behaviors [49, 50]. Network communication theories point to network analysis as the appropriate methodology to understand communication structure in complex healthcare systems.

Using these complementary frameworks, we posit that communication structure and quality interact to directly impact the quality of patient care. For example, lack of clarity and poor tone (communication quality) can impact clinician understanding of patient status and delay necessary intervention (care quality). Similarly, communication that must flow through a single lead physician has a high degree of hierarchy (communication structure), which can delay the flow of communication and necessary intervention, thus impacting patient outcomes. These interactions also impact providers, as poor communication experiences contribute to increased burnout and intentions to leave. This, in turn, further impacts patient outcomes, including safety events, mortality, and morbidity.

Our study aims (Table 1) are directly incorporated into this conceptual framework. Aim 1 focuses on the interrelationship between communication structure and quality to identify determinants of high-quality team communication. Aim 2 focuses on co-developing an appropriate multilevel intervention to target modifiable determinants of communication. Aim 3 tests the intervention’s feasibility and preliminary efficacy to improve team communication, and provider and patient outcomes in a pilot trial.

## Setting

The proposed work will be conducted in collaboration with resource-variable pediatric oncology hospitals participating in the St. Jude Global Alliance [2], an international global health research and advocacy program that aims to strengthen care for children with cancer and other catastrophic diseases through innovative interventions, education, research, and team collaboration. Currently, the alliance has collaborators from more than 300 institutions in 77 countries. Over 110 St. Jude Global Alliance hospitals have completed the CritCom survey, and many others have participated in other large studies evaluating pediatric oncology care in resource-variable settings [51–56]. Hospitals with prior CritCom participation will be eligible sites for this study. While most hospitals are located in low- and middle-income countries, several are situated in World Bank-designated high-income countries. Aligned with our previous work, we will include hospitals self-identifying as having limitations in a broad range of resources, including inadequate nursing and physician staffing to identify and manage clinical deterioration events, limited Pediatric Intensive Care Unit space to accommodate children with cancer, and patients with low socioeconomic, educational, and nutritional indicators [3, 57–59]. These challenges result in an increased frequency of deterioration events with high mortality [12, 13], and their involvement enriches our study of communication across a range of resource limitations.

### Study measures

Study measures (Table 2) align with our conceptual model, including measures of care inputs, team communication, care processes, and multilevel outcomes.

**Table 2:** Overview of study measures

Data Domain	Measures	Data Collection Methods
Communication quality	CritCom (6 domains, 30 questions)^a^	- Individual survey, distributed confidentially and electronically through REDCap [60] - Distributed to all frontline clinical staff caring for children with cancer at risk of deterioration at each participating hospital - 3 weeks for data collection with weekly reminders
Individual demographics	6 questions on role, unit worked, length of experience, sex, and hospital of employment	
Provider outcomes	Questions about burnout, intent to leave, and job satisfaction	
Hospital characteristics	Hospital type, funding, bed capacity, pediatric oncology/critical care staffing, annual new diagnoses, and region	- Collected and confirmed by local site lead
Communication structures	Social network survey using a roster-based approach asking clinicians about overall communication patterns and changes during deterioration events^a^	- Individual survey distributed confidentially through REDCap, 20–30 min. to complete - Distributed to all frontline clinical staff caring for children with cancer - 3 weeks for data collection with weekly reminders
Challenges to communication and potential communication interventions	Perspectives of clinicians on communication challenges related to hospital and individual barriers, as well as potential interventions to improve team communication during care delivery	- Semi structured interviews of frontline clinicians caring for children with cancer (physician, nurse, respiratory therapy, etc.) - Purposive sample for diversity including country income level, hospital size, professional role, etc
Intervention feasibility, acceptability, and appropriateness	After intervention implementation, measures to assess how individual clinicians view the feasibility of intervention and their ratings of how appropriate and acceptable it is for them to receive the intervention in their setting	- Individual survey, distributed confidentially through REDCap, 5 min. to complete - Distributed to all frontline clinical staff caring for children with cancer at 8 hospitals - 3 weeks per hospital for data collection, with weekly reminders
Patient Outcomes	Clinical Deterioration Events (CDEs) [13] defined as an unplanned transfer to a higher level of care (i.e., ICU), use of an ICU intervention on the ward (vasoactive infusion, invasive or noninvasive mechanical ventilation, or cardiopulmonary resuscitation), or a ward death in a patient without limitations on resuscitation	- Prospective de-identified registry of all CDEs in hospitalized pediatric oncology patients collected by the site team - For each CDE, a de-identified case report form is completed by local site leads and entered in REDCap database by a study team member - Data analysis checks for missing and incorrect values assure data quality - Pre and post intervention measurements

^a^CritCom and the network survey will be collected concurrently during Aim 1, with CritCom collected alone during Aim 3

#### Social network survey

A social network survey will be conducted among 20 hospitals. These hospitals have previously completed CritCom and all provide pediatric cancer care. All critical care and ward clinicians caring for children with will be surveyed. Questions relate to communication patterns with all other clinicians sharing the same shift, communication during deterioration, role-based communication patterns, and other questions about the structure of team communication in their setting.

#### CritCom

CritCom is a novel, valid, reliable, and multilingual measure of team communication quality in the care of children with cancer experiencing deterioration [44, 45]. CritCom was designed for use by clinical healthcare teams across different hospital settings and resource levels. Our work will continue to provide construct validity for this pragmatic measure, advancing communication measurement in different linguistic settings, which is a major scientific barrier within the field. This work will shift approaches in how team communication quality can be measured in clinical settings, resulting in opportunities to assess and intervene on communication quality.

#### Hospital characteristics

For each participating hospital, we will conduct a hospital-level survey to assess organizational characteristics, such as hospital type, funding structure, size, annual number of new childhood cancer diagnoses, and other relevant variables.

#### Implementation outcomes

During Aim 3, we will conduct surveys of the clinical team members receiving the intervention to assess perceived feasibility, acceptability, and appropriateness of this intervention in their unit/team. We will use brief, validated measures of these constructs [61].

### Expert panel

The research team will work closely with an expert panel of 20 members of the global pediatric oncology community with expertise in (1) clinical childhood cancer care in resource-variable settings, (2) healthcare team communication, and/or (3) health services delivery. The expert panel members will be specifically chosen to represent diverse geographic, contextual, and content expertise relevant to the care of children with cancer, inclusive of all WHO regions and country-income levels. This expert panel will meet quarterly, in English, to review study progress, provide feedback on findings and intervention development, participate in manuscript writing, and address any needs that arise throughout the grant period. This expert panel will also co-develop the communication intervention (Aim 2) and provide feedback throughout the pilot trial of the intervention (Aim 3).

### Aim 1: Relationship between communication structure and qualit

We will use social network methods to assess the relationship between team communication *structure* and *quality*, and how communication structures vary among hospitals providing childhood cancer care.

*Study setting and participants*. From hospitals providing childhood cancer care that completed the communication quality assessment (CritCom) between March 2023—March 2025, 20 hospitals will be identified for eligibility. Hospitals will be selected using a modified positive and negative deviance approach, targeting 10 high- and 10 low-performing hospitals as defined through the cross-sectional assessment of communication quality (CritCom scores) [44, 45]. These hospitals will be recruited to obtain variability in CritCom score, with attention paid to the region of the hospital. After identification, the site lead from prior work will be contacted. Additional hospitals will be approached should the first 20 not agree to participate. Site leads will be responsible for obtaining hospital approval for participation and guiding local data collection. Each participating hospital will receive a report that includes hospital-level strengths and weaknesses.

#### Participant recruitment

Within each participating hospital, all clinicians who care for children with cancer at risk of deterioration will be eligible. A roster of all clinicians will be developed by site leads and revised with input by the study team, as is standard in network data collection. Individuals will be consented into this aim, and their participation will conclude at the end of the social network analysis and CritCom survey. Participation will be voluntary. We will provide incentives appropriate for each region for individuals who complete the assessment.

#### Data collection

Participants will be asked to complete a single survey containing the social network and CritCom questions. The social network survey inquires about communication patterns between individuals in routine care and how these patterns change when a patient is experiencing clinical deterioration. Participants will respond to questions including their communication contacts, and frequency of communication for both routine inpatient care and deterioration events. The CritCom assessment portion measures communication quality using 5-point Likert scores across 6 domains [44, 45]. The survey will be administered through email online methods including email and WhatsApp, and participants will complete the survey online using REDCap. CritCom surveys are completed individually and averaged to obtain an institutional overall CritCom score and scores for each of six domains.

*Data management and analysis*. Data will be cleaned, managed, and analyzed in R [62]. The analysis will consist of two phases. First, we will produce network maps of team communications within each hospital. These maps will highlight how each team member communicates with other professions and disciplines at each hospital. Second, we will produce descriptive network statistics for each hospital, focusing on the interconnectedness, cross-profession connection patterns, and betweenness centralization [63–66]. Producing these statistics will identify any subgroups or other unique network patterns that arise within each of the hospital systems [64]. We are particularly interested in the network measure of betweenness centralization, which characterizes the hierarchical nature of clinical communication networks. Finally, we will build statistical models (exponential random graph model) [67] and explore the association of quality (CritCom scores) with structure (i.e., density) using the hospital-specific communication networks. This statistical model will help assess the probability that nodes (individuals) will have ties between them, allowing for the evaluation of predictors of communication based on individual characteristics and dyad attributes.

### Aim 2. Co-develop a multilevel intervention to improve team communication quality in resource-variable hospitals

We will combine perspectives of clinicians across 20 hospitals regarding modifiable determinants of team communication to co-develop a multilevel intervention to improve communication quality. This convergent mixed methods design will consist of two phases: (1) *qualitative* assessment of staff perspectives on determinants of communication quality and how this relates to provider/patient outcomes, using quantitative data from prior work and Aim 1; and (2) intervention mapping to co-develop a feasible, implementable interventions across hospitals of varying resource levels.

#### Participant recruitment

Any individual who is eligible in Aim 1 will be eligible for Aim 2. Participants will complete individual interviews to allow for honest description of potential communication struggles at their hospital and between coworkers. Participants will be sampled purposively for a range of characteristics, including country income level, hospital size, professional role, and primary unit of clinical work. We aim to enroll around 15 individuals [68] with additional recruitment using snowball sampling as necessary until we achieve information power.

#### Data collection

Interviews will be conducted using Zoom in individuals’ preferred language (English, Spanish, Portuguese, or Arabic) using native-speaking research team members. The interviewers will be individuals who are not on the participant clinical team or involved in clinical care at the hospital to facilitate honest dialogue, and interviews will be audio-recorded. There will be three main sections to the interview guide: (1) communication quality, including questions about working relationships with other professionals, the six CritCom domains of quality communication, their experience during patient care events and how they would describe effective communication in their setting; (2) communication structure, including questions such as the frequency of communication types in their setting, experience of hierarchy in the work environment, and typical processes of communication during deterioration events; and (3) interventions, including questions about perceived barriers to improving communication (modifiable determinants) and strategies that would improve communication in their setting. We will not re-collect CritCom scores, but will use the data collected in Aim 1 during the synthesis phase.

#### Analysis plan

Audio recordings will be translated and transcribed into English using a professional service [69–72]. Transcripts will be de-identified, segmented, and uploaded to MAXQDA for analysis. A qualitative analysis team will develop an initial codebook with a priori codes informed by CritCom domains, structural analysis, and the conceptual frameworks (Figs. 1 and 2), including barriers and facilitators to communication and potential interventions. Additionally, inductive codes will be developed using a constant comparative approach with iterative memoing of transcripts to allow for novel themes. Iterative revision of the codebook will be conducted across three transcripts until a final codebook is developed. Once finalized, transcripts will be independently coded by two coders, with monitoring for interrater reliability. Discrepancies will be resolved through consensus and a third adjudicator. We anticipate using two approaches: categorical coding, describing the data according to broad domains, and thematic coding, describing the relationship between concepts included in our conceptual framework.

#### Data synthesis

Convergent analysis will include quantitative output from Aim 1 with qualitative data collected in Aim 2 to understand the relationship between organizational and team determinants of communication quality and between communication structure and quality. We will use joint displays [73, 74] to understand the results of both assessments, focusing on the six domains of communication quality and structural components elucidated through the network analysis. The research team and expert panel will review these results to gain a deeper understanding of modifiable determinants of communication quality. Identified communication barriers will be reviewed and discussed to identify determinants that could be foci for intervention. Non-modifiable determinants include organizational or individual factors that cannot be changed, such as participant length of experience (hypothesized quality determinant) or building layout (hypothesized structural determinant). However, modifiable factors will be targeted for potential intervention development.

#### Intervention mapping

We will use these mixed methods results to conduct intervention mapping with members of the expert panel to co-develop a multilevel team communication intervention. Intervention mapping is an evidence-informed six-step process to develop an intervention. The expert panel and engagement with the global oncology community will inform the selection and design of an intervention to address identified team communication challenges. We will use results from Aims 1 and 2 to define the problem. We will conduct a series of workshops both in person and virtually to complete the process. Through these workshops, the team will: (1) select intervention methods and change targets that are responsive to identified needs, (2) produce key implementation strategies and attributes for intervention implementation, and (3) combine these components into a coherent multilevel intervention that is appropriate for diverse resource-variable contexts [75–77]. We will use rigorous intervention mapping procedures consistent with prior research [78–80]. At the end, we will conduct a modified Delphi to prioritize interventions and select a multilevel intervention targeting different levels of action (provider, unit, hospital). We anticipate that this multi-level intervention will focus on provider behaviors and communication structures relevant during deterioration events.

### Aim 3. Test feasibility, acceptability, appropriateness, and preliminary efficacy of a multilevel intervention to improve team communication quality in resource-variable hospitals

We will use a pilot waitlist control cluster randomized trial to assess the feasibility and preliminary efficacy of the intervention developed in Aim 2, which aims to facilitate a future hybrid implementation-effectiveness trial.

#### Study settings and recruitment

Eight hospitals will be identified from the lowest quartile of communication quality based on prior CritCom data and measured as the overall CritCom score. Previous site leads will be contacted for study participation. In partnership with the local site lead, the research team will send emails to all clinicians who meet the inclusion criteria. Identified participants will include all clinicians who care for hospitalized children with cancer at risk of deterioration. We will use marketing and materials at the hospital to increase awareness and will send up to four emails to complete the survey instruments at each time point.

#### Randomization to intervention

We will use a parallel-arm, pilot cluster-randomized wait list-controlled trial to evaluate the intervention compared to control. A wait list control is a group that does not receive an intervention during the study period, serving as an untreated comparison during the study, although they will receive the intervention after study completion [81]. This methodology was selected due to ethical concerns of restricting interventions in these settings. We will balance randomization on baseline CritCom scores to ensure distribution of communication quality across groups. Hospitals will be randomized to either the multilevel intervention or a wait list control using a 1:1 ratio via randomization protocol generated by the study biostatistician and implemented through REDCap’s randomization module. All baseline hospital and participant data will be collected prior to randomization.

#### Intervention

We will implement the multilevel intervention developed in Aim 2. We anticipate that this intervention might contain a training or series of trainings in conjunction with other systems changes, such as changing workflows and communication pathways. For this study, the intervention will be delivered by study team members but we anticipate that in the future it can be developed by local clinical team members. We will develop a full intervention manual following Aim 2 with input from the Expert Panel.

#### Training

To ensure the intervention is implemented consistently and all procedures are followed and assessed, the research team will facilitate and deliver the intervention components. A training on the intervention will be developed and refined to ensure consistent intervention delivery in the future and across hospitals. This will be consistent with our prior work [82, 83], and include information on hospital-level tailoring of the intervention. Training will also ensure consistency and accurate assessment of study outcomes.

#### Measures

We selected outcomes and covariates for this study in alignment with our prior work, expert guidance, and the conceptual framework. As a pilot study, the primary measures, in addition to assessing feasibility and recruitment in the trial, will focus on early implementation outcomes, including provider perception of the intervention's feasibility, acceptability, and appropriateness at their hospital. These will be assessed via brief, previously validated measures [61]. These items (4 questions for each measure) will be completed at 1 and 6 months following the completion of the intervention. To assess for feasibility of the study design and procedures, we will also track hospital and clinician recruitment and retention across the study.

The study will also assess preliminary effectiveness of the intervention. *Communication quality*, as measured by the CritCom survey, is the primary effectiveness outcome. This outcome will be calculated as changes in CritCom score (communication quality) from baseline to 6 months. The CritCom measure is collected at the individual level but will be analyzed as a hospital-level outcome, reflecting overall changes in hospital communication quality. Clinicians will be asked to complete the CritCom assessment and accompanying demographic questions at enrollment, one month after intervention delivery, and at the conclusion of the pilot (6 months after intervention). These time points are selected to allow for preliminary identification of when communication quality changes begin and align to the implementation outcome measures as described above.

In addition to impact on communication quality, we will explore intervention impact on secondary provider and patient effectiveness outcomes. *Provider outcomes* include burnout and retention and *patient outcomes*include clinical deterioration event mortality and safety/adverse events (cardiopulmonary arrest, cardiac arrest) [84]. These are considered exploratory as the trial is not powered to evaluate intervention effectiveness. We will evaluate these exploratory outcomes at three time points: baseline, three months after intervention, and six months after intervention. Provider outcomes will be based on self-report survey items collected along with the CritCom survey and demographic information. Patient outcomes will be reported by site leads via a de-identified prospective registry of all clinical deterioration events, defined as unplanned transfers to intensive care (ICU), the use of ICU interventions on general wards, or unplanned death on the ward [12]. All exploratory outcomes will be assessed as changes from baseline at 6 months.

#### Individual and hospital-level covariates

Participant-level demographic covariates include profession, role on the team, years working at the hospital, and sex. Hospital-level covariates include hospital type, funding structure, country income-level, staffing ratios, bed capacity, and annual new cancer diagnoses managed at the hospital per year.

#### Data collection

Implementation outcomes, communication quality (CritCom), demographic questions, and provider outcomes will be collected at the individual provider level. As described, eligible participants are clinicians who provide care to hospitalized children with cancer at risk of deterioration. Surveys will be distributed to study participants via confidential REDCap links at baseline, one month after the intervention, and at the study's completion (six months post-intervention). Hospital-level information and patient outcomes (clinical deterioration events) will be collected at the hospital level by the site lead. A de-identified report form will be completed by the site lead, or their delegate, for each deterioration event and will be entered into a REDCap database at the three data collection time points, consistent with our prior work [85–87]. Research team coordinators will complete analysis checks for missing and incomplete data and iteratively provide feedback to study teams.

#### Data analysis

Data will be managed, cleaned, and analyzed in R. As a pilot, this study is not powered to formally test effectiveness, and data analysis will focus on the feasibility of the intervention and procedures in preparation for a future trial. Data will be reported using descriptive comparisons by randomization assignment. We will describe participant and hospital characteristics overall and separately by arm. Categorical variables will be reported as N (%) and continuous variables will be reported as mean ± SD or median and interquartile range, as appropriate.

The measures of feasibility, appropriateness, and acceptability are assessed on a 5-point Likert scale. Therefore, we will report descriptive statistics of these measures prior to assessing variation via Kruskal–Wallis and Mann–Whitney tests. Data will be analyzed both at the individual and organization levels, to understand how individual and organizational factors influence intervention feasibility, appropriateness, acceptability and preliminary effectiveness. Recruitment and retention across the study will also be reported via descriptive statistics to assess feasibility of the study design and procedures for a future trial.

To evaluate the preliminary effectiveness of the intervention on perceived communication quality, we will use a linear mixed model approach where intervention is included as a fixed effect (intervention or control) along with randomization assignment, baseline score, time, and the interaction between time and intervention. We will evaluate the preliminary efficacy using a linear contrast to estimate the mean difference in communication quality (CritCom scores overall and by domain) from baseline to 6 months. To account for clustering due to individual- and hospital-level repeated measurements, we will include random terms for both in our mixed model. Exploratory provider and patient outcomes will be compared between the two randomization arms using the same mixed-effect analytic approach described for the primary effectiveness outcome. We will also conduct hospital-specific subgroup analyses to explore heterogeneity in intervention effects across hospitals.

#### Missing data

We anticipate having little missing data, due to our oversight and engagement strategies and observed response rates in our prior work [88, 89]. However, we will evaluate patterns in missing data and will analytically address missing data to avoid biasing the results. We will assume that data are missing at random, and mixed-effects models produce unbiased estimates in the presence of data missing at random [90]. We will ensure that our data meets all assumptions for statistical tests and will use data transformation or appropriate test alternatives as needed.

### Dissemination plan

Our dissemination plan incorporates different audiences, including clinicians, researchers, and funders. We will share our findings through publication and conference presentations. All study team members, including members of the Expert Panel, will participate in authoring and publishing manuscripts. During the study, we will disseminate data to participating hospitals via reports, dashboards, and annual webinars [91]. Findings will also be shared with the global pediatric oncology community regularly through the St. Jude Global Alliance. All data that are produced will be made publicly available. We will focus on translational products to amplify impact and future scale, including dissemination of public goods [92] such as our measurement tools and intervention materials. Quantitative data from this study will be made available after data collection, cleaning, and de-identification. Additionally, we will make available all codebooks and results from the qualitative components.

## Discussion

The TeamTalk study aims to develop a rigorous understanding of modifiable aspects of communication quality and structure to inform a co-developed, contextually responsive, feasible, and effective intervention to improve team communication and care for children with cancer across resource-variable hospitals globally. This study will provide a foundational understanding of modifiable determinants of team communication that impact pediatric cancer care to inform a multilevel intervention that responds to these determinants to improve childhood cancer outcomes. Ultimately, this work will improve team communication and outcomes for children with cancer in hospitals of all resource levels, thus advancing health equity globally.

This study is innovative in several ways. We will leverage implementation science and social network methods to focus on the relationship between communication structure and quality, and how they impact health outcomes, an important conceptual innovation. Additionally, this study will advance pragmatic multi-lingual measurement for team communication by using CritCom, a novel, reliable, multilingual measure of team communication. This measure was designed for clinical healthcare team use globally. This work will continue to provide construct validity for this pragmatic measure, advancing communication measurement in different linguistic settings, which is a major scientific barrier within the field. This work will shift approaches in how communication quality can be measured in clinical settings, resulting in opportunities to assess communication more broadly. Finally, this study emphasizes that improving team teamwork is a priority within cancer care [93–95], and quality of communication within teams impacts patient and provider outcomes [25, 26, 96–99]. To our knowledge, this will be the first study in pediatric oncology connecting provider- and hospital-level determinants to team communication structure and quality. Taken with prior work linking communication to outcomes, this research will provide novel empirical evidence to inform rigorous development of interventions to improve communication.

This study integrates implementation science theory to expedite the implementation of an evidence-informed, co-designed novel communication intervention in resource-variable settings. Many barriers limit implementation of new healthcare innovations [100]. On average, it takes 17 years for evidence to progress from discovery to delivery [101], and this timeframe is likely longer in low-resource settings. However, some models promote the continual improvement of care processes, which would ultimately increase high-quality healthcare. This proposal uses rapid, streamlined, participatory methods to identify local strengths along with relevant challenges to care delivery, then quickly leverage this contextual knowledge to develop and implement interventions to address identified challenges. This work thus provides important knowledge about how to effectively intervene on communication barriers by identifying communication facilitators and building on existing communicating supports to improve care. We have informed this study with implementation science principles to promote accelerated knowledge translation [102, 103] thus enhancing dissemination and the future scalability of these efforts. We expect the results of this work to advance health services research by establishing a rigorous approach for developing theoretically driven, empirically informed novel interventions that result in advances in care delivery in a range of clinical settings across all resource-levels.

Prior work demonstrates that effective team communication is a crucial determinant of quality care for children with cancer [93, 104, 105], yet little is known about how clinician communication networks vary across hospitals with different resource-levels and how these variations relate to care delivery. This study directly addresses this knowledge gap. This work will broaden our understanding of the role of healthcare team communication in pediatric cancer care through advancing the use of a novel measure of team communication quality and the development of an appropriate and feasible intervention. Ultimately, this research will design an intervention to make high-quality communication more likely, therefore having a direct impact on clinical practice and improve childhood cancer outcomes. This study will also allow for future work to evaluate both the implementation and effectiveness of this novel intervention in a larger hybrid-effectiveness trial, with a focus towards global scale-up and sustainability.

## Data Availability

Not applicable.

## Abbreviations

CDE: Clinical Deterioration Event
CritCom: A measure of team communication during pediatric deterioration
cMTS: Cancer Multiteam System framework
ICU: Intensive Care Unit
TeamTalk: The name given to this project to study team communication during healthcare delivery
WHO: World Health Organization

## References

[1] World Health Organization. Global Initiative for Childhood Cancer. . http://www.who.int/cancer/childhood-cancer/en/

[2] St. Jude Global. Accessed March 2, 2020, https://www.stjude.org/global.html

[3] Atun R, Bhakta N, Denburg A, et al. Sustainable care for children with cancer: a lancet oncology commission. Lancet Oncol. 2020;21(4):e185–224. 10.1016/s1470-2045(20)30022-x. 32240612 10.1016/S1470-2045(20)30022-X

[4] Ehrlich BS, McNeil MJ, Pham LTD, et al. Treatment-related mortality in children with cancer in low-income and middle-income countries: a systematic review and meta-analysis. Lancet Oncol. 2023;24(9):967–77. 10.1016/S1470-2045(23)00318-2. 37517410 10.1016/S1470-2045(23)00318-2 PMC10812862

[5] Jones D, Mitchell I, Hillman K, Story D. Defining clinical deterioration. Resuscitation. 2013. 10.1016/j.resuscitation.2013.01.013. 23376502 10.1016/j.resuscitation.2013.01.013

[6] Padilla RM, Mayo AM. Clinical deterioration: a concept analysis. J Clin Nurs. 2018;27(7–8):1360–8. 10.1111/jocn.14238. 29266536 10.1111/jocn.14238

[7] Ceppi F, Antillon F, Pacheco C, et al. Supportive medical care for children with acute lymphoblastic leukemia in low- and middle-income countries. Expert Rev Hematol. 2015;8(5):613–26. 10.1586/17474086.2015.1049594. 26013005 10.1586/17474086.2015.1049594

[8] Duke T, Cheema B. Paediatric emergency and acute care in resource poor settings. J Paediatr Child Health. 2016;52(2):221–6. 10.1111/jpc.13105. 27062627 10.1111/jpc.13105

[9] Dray E, Mack R, Soberanis D, Rodriguez-Galindo C, Agulnik A. Beyond supportive care: a collaboration to improve the intensive care management of critically Ill Pediatric oncology patients in resource-limited settings. Pediatr blood cancer. 2017;64 Suppl 3(Suppl 3)10.1002/pbc.26772

[10] Friedrich P, Ortiz R, Fuentes S, et al. Barriers to effective treatment of pediatric solid tumors in middle-income countries: can we make sense of the spectrum of nonbiologic factors that influence outcomes? Cancer. 2014;120(1):112–25. 10.1002/cncr.28339. 24132910 10.1002/cncr.28339 PMC3934757

[11] Rodriguez-Galindo C, Friedrich P, Morrissey L, Frazier L. Global challenges in pediatric oncology. Curr Opin Pediatr. 2013;25(1):3–15. 10.1097/MOP.0b013e32835c1cbe. 23295716 10.1097/MOP.0b013e32835c1cbe

[12] Agulnik A, Cárdenas A, Carrillo AK, et al. Clinical and organizational risk factors for mortality during deterioration events among pediatric oncology patients in Latin America: a multicenter prospective cohort. Cancer. 2021. 10.1002/cncr.33411. 33524166 10.1002/cncr.33411 PMC8248122

[13] Agulnik A, Robles-Murguia M, Chen Y, et al. Multilevel mortality risk factors among pediatric hematology-oncology patients with deterioration. Cancer. 2025;131(8):e35818. 10.1002/cncr.35818. 40193253 10.1002/cncr.35818 PMC11975202

[14] Agulnik A M-THPLTDCYCAKC-AARAGGMZTVD. Effect of paediatric early warning systems (PEWS) implementation on clinical deterioration event mortality among children with cancer in resource-limited hospitals in Latin America: a prospective, multicentre cohort study. Lancet Oncology. 2023;10.1016/S1470-2045(23)00285-1 10.1016/S1470-2045(23)00285-1 PMC10727097 37433316

[15] Reader TW, Flin R, Mearns K, Cuthbertson BH. Interdisciplinary communication in the intensive care unit. Br J Anaesth. 2007;98(3):347–52. 10.1093/bja/ael372. 17272386 10.1093/bja/ael372

[16] Gluyas H. Effective communication and teamwork promotes patient safety. Nurs Stand. 2015;29(49):50–7. 10.7748/ns.29.49.50.e10042. 26243123 10.7748/ns.29.49.50.e10042

[17] Olde Bekkink M, Farrell SE, Takayesu JK. Interprofessional communication in the emergency department: residents’ perceptions and implications for medical education. Int J Med Educ. 2018;9:262–70. 10.5116/ijme.5bb5.c111. 30368487 10.5116/ijme.5bb5.c111 PMC6387781

[18] The Joint Commission. http://www.jointcommission.org/sentinel_event.aspx

[19] O’Brien A, O’Reilly K, Dechen T, et al. Redesigning rounds in the ICU: standardizing key elements improves interdisciplinary communication. Jt Comm J Qual Patient Saf. 2018;44(10):590–8. 10.1016/j.jcjq.2018.01.006. 30064951 10.1016/j.jcjq.2018.01.006

[20] Rothschild JM, Landrigan CP, Cronin JW, et al. The critical care safety study: the incidence and nature of adverse events and serious medical errors in intensive care. Crit Care Med. 2005;33(8):1694–700. 10.1097/01.ccm.0000171609.91035.bd. 16096443 10.1097/01.ccm.0000171609.91035.bd

[21] Foronda C, MacWilliams B, McArthur E. Interprofessional communication in healthcare: an integrative review. Nurse Educ Pract. 2016;19:36–40. 10.1016/j.nepr.2016.04.005. 27428690 10.1016/j.nepr.2016.04.005

[22] Inadequate hand-off communication. Sentinel Event Alert. Sep 12 2017;(58):1–6. 28914519

[23] Smith IJ, Joint Commission Resources I. The Joint Commission Guide to Improving Staff Communication. Joint Commission Resources; 2005.

[24] Ranelli PL, Biss J. Physicians’ perceptions of communication with and responsibilities of pharmacists. J Am Pharm Assoc (Wash). 2000;40(5):625–30. 10.1016/s1086-5802(16)31102-0. 11029843 10.1016/s1086-5802(16)31102-0

[25] Ghaferi AA, Dimick JB. Importance of teamwork, communication and culture on failure-to-rescue in the elderly. Br J Surg. 2016;103(2):e47-51. 10.1002/bjs.10031. 26616276 10.1002/bjs.10031 PMC4715639

[26] von Knorring M, Griffiths P, Ball J, Runesdotter S, Lindqvist R. Patient experience of communication consistency amongst staff is related to nurse-physician teamwork in hospitals. Nurs Open. 2020;7(2):613–7. 10.1002/nop2.431. 32089859 10.1002/nop2.431 PMC7024626

[27] Ratelle JT, Kelm DJ, Halvorsen AJ, West CP, Oxentenko AS. Predicting and communicating risk of clinical deterioration: an observational cohort study of internal medicine residents. J Gen Intern Med. 2015;30(4):448–53. 10.1007/s11606-014-3114-4. 25451991 10.1007/s11606-014-3114-4 PMC4370993

[28] Endacott R, Kidd T, Chaboyer W, Edington J. Recognition and communication of patient deterioration in a regional hospital: a multi-methods study. Aust Crit Care. 2007;20(3):100–5. 10.1016/j.aucc.2007.05.002. 17627836 10.1016/j.aucc.2007.05.002

[29] Busari JO, Moll FM, Duits AJ. Understanding the impact of interprofessional collaboration on the quality of care: a case report from a small-scale resource limited health care environment. J Multidiscip Healthc. 2017;10:227–34. 10.2147/jmdh.S140042. 28652761 10.2147/JMDH.S140042 PMC5472431

[30] Blakeney EA, Chu F, White AA, et al. A scoping review of new implementations of interprofessional bedside rounding models to improve teamwork, care, and outcomes in hospitals. J Interprof Care. 2021;10:1–16. 10.1080/13561820.2021.1980379. 10.1080/13561820.2021.1980379 PMC8994791 34632913

[31] Reimer N, Herbener L. Round and round we go: rounding strategies to impact exemplary professional practice. Clin J Oncol Nurs. 2014. 10.1188/14.CJON.18-06AP. 25305021 10.1188/14.CJON.18-06AP

[32] El Saghir NS, Keating NL, Carlson RW, Khoury KE, Fallowfield L. Tumor boards: optimizing the structure and improving efficiency of multidisciplinary management of patients with cancer worldwide. Am Soc Clin Oncol Educ Book. 2014:e461–6. 10.14694/EdBook_AM.2014.34.e461 10.14694/EdBook_AM.2014.34.e461 24857140

[33] Specchia ML, Frisicale EM, Carini E, et al. The impact of tumor board on cancer care: evidence from an umbrella review. BMC Health Serv Res. 2020;20:1–14. 10.1186/s12913-020-4930-3 PMC6995197 32005232

[34] Bagnasco A, Tubino B, Piccotti E, et al. Identifying and correcting communication failures among health professionals working in the emergency department. Int Emerg Nurs. 2013;21(3):168–72. 10.1016/j.ienj.2012.07.005. 23207054 10.1016/j.ienj.2012.07.005

[35] Epstein NE. Multidisciplinary in-hospital teams improve patient outcomes: a review. Surg Neurol Int. 2014;5(Suppl 7):S295-303. 10.4103/2152-7806.139612. 25289149 10.4103/2152-7806.139612 PMC4173201

[36] Bowsher G, Papamichail A, El Achi N, et al. A narrative review of health research capacity strengthening in low and middle-income countries: lessons for conflict-affected areas. Glob Health. 2019;15(1):23. 10.1186/s12992-019-0465-y. 10.1186/s12992-019-0465-y PMC6434620 30914049

[37] Edwards N, Barker PM. The importance of context in implementation research. JAIDS J Acquir Immune Defic Syndr. 2014. 10.1097/QAI.0000000000000322. 25310123 10.1097/QAI.0000000000000322

[38] Mielke J, De Geest S, Zúñiga F, et al. Understanding dynamic complexity in context—Enriching contextual analysis in implementation science from a constructivist perspective. Hypothesis and Theory. Frontiers in Health Services. 2022-July-22 2022;210.3389/frhs.2022.953731 10.3389/frhs.2022.953731 PMC10012673 36925847

[39] Ridde V, Pérez D, Robert E. Using implementation science theories and frameworks in global health. BMJ Glob Health. 2020;5(4):e002269. 10.1136/bmjgh-2019-002269. 32377405 10.1136/bmjgh-2019-002269 PMC7199704

[40] Chollette V, Doose M, Sanchez J, Weaver SJ. Teamwork competencies for interprofessional cancer care in multiteam systems: a narrative synthesis. J Interprof Care. 2022;36(4):617–25. 10.1080/13561820.2021.1932775. 34311658 10.1080/13561820.2021.1932775

[41] Verhoeven DC, Chollette V, Lazzara EH, Shuffler ML, Osarogiagbon RU, Weaver SJ. The anatomy and physiology of teaming in cancer care delivery: a conceptual framework. JNCI J Natl Cancer Inst. 2020;113(4):360–70. 10.1093/jnci/djaa166. 10.1093/jnci/djaa166 PMC8599835 33107915

[42] Algaba E, van den Brink R, Dietz C. Network Structures with Hierarchy and Communication. J Optimization Theory Appl. 2018;179(1):265–82. 10.1007/s10957-018-1348-8. (2018/10/01).

[43] Perkins JM, Subramanian SV, Christakis NA. Social networks and health: a systematic review of sociocentric network studies in low- and middle-income countries. Soc Sci Med. 2015;125:60–78. 10.1016/j.socscimed.2014.08.019. 25442969 10.1016/j.socscimed.2014.08.019 PMC5690563

[44] Lawton R, Taylor N, Clay-Williams R, Braithwaite J. Positive deviance: a different approach to achieving patient safety. BMJ Qual Saf. 2014;23(11):880–3. 25049424 10.1136/bmjqs-2014-003115 PMC4215344

[45] Rose AJ, McCullough MB. A practical guide to using the Positive Deviance method in health services research. Health Serv Res. 2017;52(3):1207–22. 10.1111/1475-6773.12524. 27349472 10.1111/1475-6773.12524 PMC5441507

[46] P. C. Understanding Complex Systems. Handbook of Systems and Complexity in Health. Springer; 2013.

[47] Burnes B. Complexity theories and organizational change. Int J Manag Rev. 2005;7(2):73–90. 10.1111/j.1468-2370.2005.00107.x.

[48] Monge PR, Contractor NS, Contractor PS, Peter R, Noshir S. Theories of communication networks. USA: Oxford University Press; 2003.

[49] Phelan S. A note on the correspondence between complexity and systems theory. Syst Pract Action Res. 1999;12(3):237–46. 10.1023/A:1022495500485.

[50] Amagoh F. Perspectives on organizational change: systems and complexity theories. Innov J. 2008;13(3):1–14.

[51] Graetz D, Agulnik A, Ranadive R, et al. Global effect of the COVID-19 pandemic on paediatric cancer care: a cross-sectional study. Lancet Child Adolesc Health. 2021;5(5):332–40. 10.1016/S2352-4642(21)00031-6. 33675698 10.1016/S2352-4642(21)00031-6 PMC7929816

[52] Ehrlich BS, Movsisyan N, Batmunkh T, et al. Barriers to the early integration of palliative care in pediatric oncology in 11 Eurasian countries. Cancer. 2020. 10.1002/cncr.33151. 32813913 10.1002/cncr.33151 PMC7981844

[53] Ehrlich BS, Movsisyan N, Batmunkh T, et al. A multicountry assessment in Eurasia: alignment of physician perspectives on palliative care integration in pediatric oncology with World Health Organization guidelines. Cancer. 2020;126(16):3777–87. 10.1002/cncr.33001. 32530519 10.1002/cncr.33001 PMC7385991

[54] McNeil MJ, Ehrlich BS, Wang H, et al. Physician perceptions of palliative care for children with cancer in Latin America. JAMA Netw Open. 2022;5(3):e221245–e221245. 10.1001/jamanetworkopen.2022.1245. 35258577 10.1001/jamanetworkopen.2022.1245 PMC8905380

[55] Agulnik A, Gonzalez Ruiz A, Muniz-Talavera H, et al. Model for regional collaboration: Successful strategy to implement a pediatric early warning system in 36 pediatric oncology centers in Latin America. Cancer. 2022;10.1002/cncr.34427 10.1002/cncr.34427 PMC9828186 36161436

[56] Arias AV, Sakaan FM, Puerto-Torres M, et al. Development and pilot testing of PROACTIVE: a pediatric onco-critical care capacity and quality assessment tool for resource-limited settings. Cancer Med. 2023. 10.1002/cam4.5395. 36324249 10.1002/cam4.5395 PMC10028058

[57] Rodriguez-Galindo C, Friedrich P, Alcasabas P, et al. Toward the cure of all children with cancer through collaborative efforts: pediatric oncology as a global challenge. J Clin Oncol. 2015;33(27):3065–73. 10.1200/jco.2014.60.6376. 26304881 10.1200/JCO.2014.60.6376 PMC4979198

[58] Diaz F, Carvajal C, González-Dambrauskas S, et al. Abstract O-44: organizational characteristics and resources in Latin-American pediatric intensive care units. Preliminary report of REAL-CIP (realidad en America Latina de cuidados intensivos pediátricos) study. Pediatr Crit Care Med. 2018;19(6S):19. 10.1097/01.pcc.0000537386.99956.bf.

[59] Campos-Mino S, Sasbon JS, von Dessauer B. [Pediatric intensive care in Latin America]. Medicina intensiva. 2012;36(1):3–10. Los cuidados intensivos pediatricos en Latinoamerica. 10.1016/j.medin.2011.07.004 10.1016/j.medin.2011.07.004 21906846

[60] Harris PA, Taylor R, Thielke R, Payne J, Gonzalez N, Conde JG. Research electronic data capture (REDCap)–a metadata-driven methodology and workflow process for providing translational research informatics support. J Biomed Inform. 2009;42(2):377–81. 10.1016/j.jbi.2008.08.010. 18929686 10.1016/j.jbi.2008.08.010 PMC2700030

[61] Weiner BJ, Lewis CC, Stanick C, et al. Psychometric assessment of three newly developed implementation outcome measures. Implement Sci. 2017;12(1):108. 10.1186/s13012-017-0635-3. 28851459 10.1186/s13012-017-0635-3 PMC5576104

[62] Team RC. R: A language and environment for statistical computing. 2013;

[63] Knoke D, Yang S. Social network analysis. SAGE publications; 2019.

[64] Luke DA. A user's guide to network analysis in R. vol 72. Springer; 2015.

[65] Wasserman S, Faust K. Social network analysis: Methods and applications. 1994;

[66] Wellman B. Network analysis: some basic principles. Sociol Theory. 1983. 10.2307/202050.

[67] Harris JK. An introduction to exponential random graph modeling. vol 173. Sage Publications; 2013.

[68] Hennink M, Kaiser BN. Sample sizes for saturation in qualitative research: a systematic review of empirical tests. Soc Sci Med. 2022;292:114523. 10.1016/j.socscimed.2021.114523. 34785096 10.1016/j.socscimed.2021.114523

[69] Agulnik A, Ferrara G, Puerto-Torres M, et al. Assessment of barriers and enablers to implementation of a pediatric early warning system in resource-limited settings. JAMA Netw Open. 2022;5(3):e221547. 10.1001/jamanetworkopen.2022.1547. 35262714 10.1001/jamanetworkopen.2022.1547 PMC8908074

[70] Agulnik A, Malone S, Puerto-Torres M, et al. Reliability and validity of a Spanish-language measure assessing clinical capacity to sustain Paediatric Early Warning Systems (PEWS) in resource-limited hospitals. BMJ Open. 2021;11(10):e053116. 10.1136/bmjopen-2021-053116. 34670767 10.1136/bmjopen-2021-053116 PMC8529978

[71] Graetz D, Kaye EC, Garza M, et al. Qualitative Study of Pediatric Early Warning Systems’ Impact on Interdisciplinary Communication in Two Pediatric Oncology Hospitals With Varying Resources. JCO Global Oncology. 2020;(6):1079–1086. 10.1200/GO.20.00163 2020/11/01 10.1200/GO.20.00163 PMC7392735 32673079

[72] Graetz DE, Giannars E, Kaye EC, et al. Clinician emotions surrounding pediatric oncology patient deterioration. Front Oncol. 2021;11:626457. 10.3389/fonc.2021.626457. 33718195 10.3389/fonc.2021.626457 PMC7947818

[73] Guetterman TC, Fetters MD, Creswell JW. Integrating quantitative and qualitative results in health science mixed methods research through joint displays. Annals Fam Med. 2015;13(6):554–61. 10.1370/afm.1865 PMC4639381 26553895

[74] Kahwati LC, Kane HL. Qualitative comparative analysis in mixed methods research and evaluation. vol 6. Sage Publications; 2018.

[75] Bartholomew LK, Parcel GS, Kok G. Intervention mapping: a process for developing theory and evidence-based health education programs. Health Educ Behav. 1998;25(5):545–63. 9768376 10.1177/109019819802500502

[76] Fernandez ME, Ten Hoor GA, Van Lieshout S, et al. Implementation mapping: using intervention mapping to develop implementation strategies. Front Public Health. 2019;7:158. 31275915 10.3389/fpubh.2019.00158 PMC6592155

[77] Kok G, Gottlieb NH, Peters G-JY, et al. A taxonomy of behaviour change methods: an intervention mapping approach. Health Psychol Rev. 2016;10(3):297–312. 26262912 10.1080/17437199.2015.1077155 PMC4975080

[78] Durks D, Fernandez-Llimos F, Hossain LN, Franco-Trigo L, Benrimoj SI, Sabater-Hernández D. Use of intervention mapping to enhance health care professional practice: a systematic review. Health Educ Behav. 2017;44(4):524–35. 28580805 10.1177/1090198117709885

[79] Eldredge LKB, Markham CM, Ruiter RA, Fernández ME, Kok G, Parcel GS. Planning health promotion programs: an intervention mapping approach. John Wiley & Sons; 2016.

[80] Garba RM, Gadanya MA. The role of intervention mapping in designing disease prevention interventions: a systematic review of the literature. PLoS ONE. 2017;12(3):e0174438. 28358821 10.1371/journal.pone.0174438 PMC5373531

[81] Elliott SA, Brown JS. What are we doing to waiting list controls? Behav Res Ther. 2002;40(9):1047–52. 10.1016/s0005-7967(01)00082-1. 12296489 10.1016/s0005-7967(01)00082-1

[82] Malone S, McKay VR, Krucylak C, et al. A cluster randomized stepped-wedge trial to de-implement unnecessary post-operative antibiotics in children: the optimizing perioperative antibiotic in children (OPerAtiC) trial. Implement Sci. 2021;16(1):1–11. 33741048 10.1186/s13012-021-01096-1 PMC7980649

[83] Agulnik A, Gonzalez Ruiz A, Muniz-Talavera H, et al. Model for regional collaboration: successful strategy to implement a pediatric early warning system in 36 pediatric oncology centers in Latin America. Cancer. 2022;128(22):4004–16. 10.1002/cncr.34427. 36161436 10.1002/cncr.34427 PMC9828186

[84] Abu-Rish Blakeney E, Baird J, Beaird G, et al. How and why might interprofessional patient- and family-centered rounds improve outcomes among healthcare teams and hospitalized patients? A conceptual framework informed by scoping and narrative literature review methods. Mini Review. Frontiers in Medicine. 2023-October-11 2023;1010.3389/fmed.2023.1275480 10.3389/fmed.2023.1275480 PMC10598853 37886364

[85] Agulnik A, Cardenas A, Carrillo AK, et al. Clinical and organizational risk factors for mortality during deterioration events among pediatric oncology patients in Latin America: a multicenter prospective cohort. Cancer. 2021;127(10):1668–78. 10.1002/cncr.33411. 33524166 10.1002/cncr.33411 PMC8248122

[86] Agulnik A, Forbes PW, Stenquist N, Rodriguez-Galindo C, Kleinman M. Validation of a pediatric early warning score in hospitalized pediatric oncology and hematopoietic stem cell transplant patients. Pediatr Crit Care Med. 2016;17(4):e146–53. 10.1097/PCC.0000000000000662. 26914628 10.1097/PCC.0000000000000662

[87] Agulnik A, Soberanis Vasquez DJ, García Ortiz JE, et al. Successful Implementation of a Pediatric Early Warning Score in a Resource-Limited Pediatric Oncology Hospital in Guatemala. J Glob Oncol 2016;2(3_suppl):60s-60s. 10.1200/JGO.2016.003871 2016/06/01

[88] McKay V, Chen Y, Prewitt K, et al. Connecting clinical capacity and intervention sustainability in resource-variable pediatric oncology centers in Latin America. Glob Implement Res Appl. 2024;4(1):102–15. 10.1007/s43477-023-00106-2. 38566954 10.1007/s43477-023-00106-2 PMC10987010

[89] Rivera J, Malone S, Puerto-Torres M, et al. Critcom: assessment of quality of interdisciplinary communication around deterioration in pediatric oncologic patients. Front Oncol. 2023. 10.3389/fonc.2023.1207578. (Original Research). 37886167 10.3389/fonc.2023.1207578 PMC10598383

[90] Snijders T, Bosker R. Multilevel analysis: an introduction to basic and advanced multilevel modeling. http://lst-iiepiiep-unescoorg/cgi-bin/wwwi32exe/[in=epidoc1in]/?t2000=013777/(100). 01/01 1999;

[91] Counts L, Rivera J, Wiphatphumiprates P, et al. Assessment of the quality of interdisciplinary communication (CritCom): evaluation and refinement of a center summary report. Front Oncol. 2024;14:1384597. 10.3389/fonc.2024.1384597. 38988704 10.3389/fonc.2024.1384597 PMC11234842

[92] Brownson RC, Eyler AA, Harris JK, Moore JB, Tabak RG. Getting the word out: new approaches for disseminating public health science. J Public Health Manag Pract. 2018;24(2):102–11. 10.1097/PHH.0000000000000673. 28885319 10.1097/PHH.0000000000000673 PMC5794246

[93] Jain AK, Fennell ML, Chagpar AB, Connolly HK, Nembhard IM. Moving toward improved teamwork in cancer care: the role of psychological safety in team communication. J Oncol Pract. 2016;12(11):1000–11. 10.1200/JOP.2016.013300. (2016/11/01). 27756800 10.1200/JOP.2016.013300

[94] National Academies of Sciences E, Medicine. Developing and sustaining an effective and resilient oncology careforce: Proceedings of a workshop. National Academies Press; 2019. 31577398

[95] Mathieu JE. Teams, teaming, and complex systems in cancer care. JCO Oncol Pract. 2023;19(1):6–9. 10.1200/op.22.00650. 36516363 10.1200/OP.22.00650

[96] Lingard L, Regehr G, Orser B, et al. Evaluation of a preoperative checklist and team briefing among surgeons, nurses, and anesthesiologists to reduce failures in communication. Arch Surg. 2008;143(1):12–7. 10.1001/archsurg.2007.21. (discussion 18). 18209148 10.1001/archsurg.2007.21

[97] Manias E, Geddes F, Watson B, Jones D, Della P. Communication failures during clinical handovers lead to a poor patient outcome: lessons from a case report. SAGE Open Med Case Rep. 2015;3:2050313X15584859. 10.1177/2050313X15584859. 27489689 10.1177/2050313X15584859 PMC4857297

[98] Ong MS, Coiera E. A systematic review of failures in handoff communication during intrahospital transfers. Jt Comm J Qual Patient Saf. 2011;37(6):274–84. 10.1016/s1553-7250(11)37035-3. 21706987 10.1016/s1553-7250(11)37035-3

[99] Umberfield E, Ghaferi AA, Krein SL, Manojlovich M. Using incident reports to assess communication failures and patient outcomes. Jt Comm J Qual Patient Saf. 2019;45(6):406–13. 10.1016/j.jcjq.2019.02.006. 30935883 10.1016/j.jcjq.2019.02.006 PMC6590519

[100] Damschroder LJ, Aron DC, Keith RE, Kirsh SR, Alexander JA, Lowery JC. Fostering implementation of health services research findings into practice: a consolidated framework for advancing implementation science. Implement Sci. 2009;4(1):50. 10.1186/1748-5908-4-50. 19664226 10.1186/1748-5908-4-50 PMC2736161

[101] Morris ZS, Wooding S, Grant J. The answer is 17 years, what is the question: understanding time lags in translational research. J R Soc Med. 2011;104(12):510–20. 22179294 10.1258/jrsm.2011.110180 PMC3241518

[102] Colquhoun HL, Squires JE, Kolehmainen N, Fraser C, Grimshaw JM. Methods for designing interventions to change healthcare professionals’ behaviour: a systematic review. Implement Sci. 2017;12(1):30. 10.1186/s13012-017-0560-5. 28259168 10.1186/s13012-017-0560-5 PMC5336662

[103] Ramsey AT, Proctor EK, Chambers DA, et al. Designing for accelerated translation (DART) of emerging innovations in health. J Clin Transl Sci. 2019;3(2–3):53–8. 10.1017/cts.2019.386. 31528365 10.1017/cts.2019.386 PMC6746422

[104] Emold C, Schneider N, Meller I, Yagil Y. Communication skills, working environment and burnout among oncology nurses. Eur J Oncol Nurs. 2011;15(4):358–63. 10.1016/j.ejon.2010.08.001. 20863757 10.1016/j.ejon.2010.08.001

[105] Graetz DE, Chen Y, Devidas M, et al. Interdisciplinary care of pediatric oncology patients in Central America and the Caribbean. Cancer. 2020. 10.1002/cncr.33339. 33237591 10.1002/cncr.33339

